# Unbiased Estimate of Synonymous and Nonsynonymous Substitution Rates with Nonstationary Base Composition

**DOI:** 10.1093/molbev/msx308

**Published:** 2017-12-06

**Authors:** Laurent Guéguen, Laurent Duret

**Affiliations:** Laboratoire de Biologie et Biométrie Évolutive, CNRS UMR 5558, Université Claude Bernard Lyon 1—Université de Lyon, Villeurbanne, France

**Keywords:** selection, synonymous substitutions, nonsynonymous substitutions, stochastic mapping

## Abstract

The measurement of synonymous and nonsynonymous substitution rates (d*S* and d*N*) is useful for assessing selection operating on protein sequences or for investigating mutational processes affecting genomes. In particular, the ratio $\frac{dN}{dS}$ is expected to be a good proxy for *ω*, the ratio of fixation probabilities of nonsynonymous mutations relative to that of neutral mutations. Standard methods for estimating d*N*, d*S*, or *ω* rely on the assumption that the base composition of sequences is at the equilibrium of the evolutionary process. In many clades, this assumption of stationarity is in fact incorrect, and we show here through simulations and analyses of empirical data that nonstationarity biases the estimate of d*N*, d*S*, and *ω*. We show that the bias in the estimate of *ω* can be fixed by explicitly taking into consideration nonstationarity in the modeling of codon evolution, in a maximum likelihood framework. Moreover, we propose an exact method for estimating d*N* and d*S* on branches, based on stochastic mapping, that can take into account nonstationarity. This method can be directly applied to any kind of codon evolution model, as long as neutrality is clearly parameterized.

## Introduction

The intensity and direction of selection operating on protein sequences can be evaluated by comparing the probability of fixation of nonsynonymous mutations to that of neutral mutations (Yang and Bielawski 2000). The ratio of fixation probabilities of nonsynonymous versus neutral mutations (denoted by *ω*) is commonly estimated by comparing nonsynonymous versus synonymous substitutions rates (denoted, respectively, by d*N* and d*S*): under the assumption that selection on synonymous sites is negligible, the ratio $\frac{dN}{dS}$ is expected to be a proxy for *ω*, and therefore, to be informative on selective regimes on protein-coding sequences. Furthermore, besides the ratio *ω*, estimates of synonymous and nonsynonymous substitution rates can also be useful in themselves. For instance, both rates can be used as molecular clocks (Kumar 2005). Moreover, under the assumption of neutrality, d*S* can be informative about variation in mutation rates both among lineages (Kumar and Subramanian 2002) and within genomes (Wolfe et al. 1989).

Substitution rates (d*N* and d*S*) are expressed in terms of number of (non)synonymous substitutions per (non)synonymous site. One important issue is therefore to quantify the number of (non)synonymous sites. Historically, the first methods developed to estimate d*S* and d*N* directly compared sequences to count the numbers of synonymous and nonsynonymous substitutions, and used elaborate formulas to account for the “per (non)substitution site” feature (Li et al. 1985; Nei and Gojobori 1986).

Subsequent methods relied on sequence alignments in a phylogenetic context, and probabilistic codon-based substitution models with *ω* as a parameter (Goldman and Yang 1994; Yang and Nielsen 2000; Guindon et al. 2004; Kosakovsky Pond et al. 2005; Yang 2007). The maximum likelihood approach thus provides estimates of *ω* and, through ancestral sequence reconstruction, inferences of d*N* and *dS*: on each branch, the number of (non)synonymous substitutions is estimated, and to take into consideration the “per (non)synonymous site” feature, the expected numbers of (non)synonymous neutral substitutions are estimated by applying the same model but without selection (i.e., the equivalent neutral model) (Goldman and Yang 1994; Yang and Nielsen 2000; Kosakovsky Pond and Frost 2005).

But up to now, programs used to compute d*N* and d*S* have two drawbacks. First, they propose approximate computations of the numbers needed, for the counting of effective (non)synonymous substitutions as well as for the normalization “per (non)synonymous site.” For example, in an article published in 2005, Kosakovsky-Pond and Frost consider the most parsimonious substitution scenarios between expected ancestral states at top and bottom of the branches, and compute which part of each scenario is synonymous or not (Kosakovsky Pond and Frost 2005). Afterwards, they use an inferred model and its neutral equivalent to estimate d*N* and d*S*. However, choosing a given substitution scenario (the most parsimonious, or even the most likely one) results in many other scenarios being ignored, especially as the branch gets longer and selection gets weaker.

Second, these programs assume stationarity in the modeling of the data, that is, assume that codon frequencies are constant all along the evolutionary process. It is now well established that in many cases this assumption is false. For instance, changes in GC-content are frequently observed in bacteria, notably during the reductive genome evolution of endosymbionts such as *Buchnera aphidicola* (van Ham et al. 2003; Moran et al. 2008; Moran 1996; Prez-Brocal et al. 2006), but also in free-living organisms such as *Prochlorococcus marinus* (Rocap et al. 2003; Dufresne et al. 2005; Yu et al. 2012; Dufresne et al. 2003; Paul et al. 2010). In mammals, genomic landscapes are characterized by large-scale variation in GC-content along chromosomes (the so-called isochores) (Bernardi et al. 1985). The processes driving the evolution of isochores fluctuate over time (Romiguier et al. 2010) and also spatially. For instance, in the human lineage, the GC-content of genes located in GC-rich isochores is decreasing, whereas those in GC-poor regions are at equilibrium (Duret and Arndt 2008). These changes in GC-content affect both codon (Wernegreen and Moran 1999; Moran 1996) and amino-acid (Mouchiroud et al. 1991; Wernegreen and Moran 1999; Itoh et al. 2002; Moran et al. 2008) frequencies. But importantly, the intragenomic variance in GC-content is much higher at synonymous than at nonsynonymous codon positions, and hence the nonstationarity affects differently d*S* and d*N* (Galtier et al. 2009; Bolivar et al. 2016).

In this article, we illustrate through simulations how assuming stationarity leads to a systematic bias in d*N*, d*S*, and $\frac{dN}{dS}$ estimates, and we show that this bias can be properly removed if stationarity is not assumed. Next, we introduce a new method, *mapdNdS*, based on stochastic mapping, for an accurate estimate of d*N* and d*S*. Instead of choosing a given scenario between pairs of ancestral states on branches, this method integrates over all possible scenarios, in accordance with their probability given the model and the length of the branch, to compute d*N* and d*S* more precisely, following the definition given by Kosakovsky Pond and Frost (2005). We implemented *mapdNdS* in bio ++ libraries (Guéguen et al. 2013), so that it can be used without any stationarity or process homogeneity constraints, and can give access to branch- and/or site-specific estimates. Using this method, we explore the bias induced by the assumption of stationarity on the estimates of d*N*, d*S*, and $\frac{dN}{dS}$, and show that this problem of bias is resolved with our method. Finally, an application of this method and the importance of taking nonstationarity into account are illustrated on a set of orthologous primate genes.

## New Approaches

Stochastic mapping is a way to infer substitution events based on probabilistic modeling estimates. In 2002, Rasmus Nielsen proposed a bayesian approach to map substitution events on the branches of a phylogenetic tree, given a probabilistic substitution model (Nielsen 2002). Since then, much theoretical and computational work has been done to describe accurately the substitution process along a phylogenetic tree, given a probabilistic model and a sequence alignment (Dutheil et al. 2005; Minin and Suchard 2008; Hobolth and Stone 2009).

This work is based on computing the expected number of substitution events of a given category along a branch. These estimates are conditioned by the states at both ends of this branch. Moreover, Minin and Suchard have proposed a way to compute the expected time spent in a given state on this branch, under the same conditions (Minin and Suchard 2008). With real data, the sequences on the ancestral nodes are not known, but it is possible to compute the posterior expectations on each branch given the data and the substitution process (Romiguier et al. 2012).

Hereafter, we use this methodology specifically on two categories of events: synonymous substitutions and nonsynonymous substitutions. Similarly to the “per (non)synonymous site” normalization for d*N* and d*S*, the expected numbers of substitution events on a branch have to be corrected given the changing ancestral sequence all along the branch. O’Brien et al. (2009) described this problem and showed that even if a simple factor 3 is used as a proxy for the relative proportion of nonsynonymous over synonymous sites, results based on mapping counts are more accurate than with previous methods of d*N* and d*S* estimate, such as the one described by Yang and Nielsen (2000). Here, we propose a method based on stochastic mapping and on the use of a neutral model, similar to previously described models (Yang and Nielsen 2000; Kosakovsky Pond and Frost 2005), to normalize the expected counts of each category.

## Results

### Assessment on Simulated Data

On all data, we inferred the most likely model, which gave the estimate of *ω*, and then we used our approach to estimate d*N*, d*S*, and $\frac{dN}{dS}$.

**Fig. 1.**
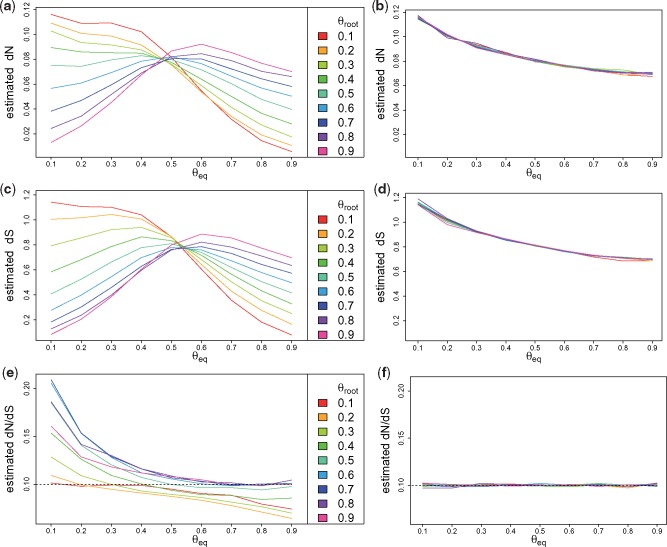
Estimates of d*N*, d*S*, and $\frac{dN}{dS}$ with a stationary model (left) and with a nonstationary model (right), on simulated data with changing G + C content and ω=0.1. *θ*_root_: G + C frequency in the root sequence. *θ*_eq_: G + C equilibrium frequency of the simulation model.

When *ω* is estimated with a stationary model, decreasing G + C content along the tree results in a systematic overestimate of *ω*, and increasing G + C content results in a systematic underestimate of *ω* (see supplementary fig. S1*a*, Supplementary Material online). We observe similar biases in the estimates of $\frac{dN}{dS}$ estimated with stochastic mapping (fig. 1*e*).

These under or overestimates can lead to false qualitative interpretation of selection, as dubious positive selection can be inferred in case of decreasing GC-content, or dubious negative selection in case of increasing GC-content (as illustrated in simulations with neutral and nearly neutral models, see supplementary figs. S2–S4, Supplementary Material online). We also performed simulations where the G + C content of one specific codon position evolved, and the two others remained stationary with 50% G + C. Again, we observed that models that assume stationarity lead to biased estimates of substitution rates and of *ω* (see supplementary figs. S6–S8, Supplementary Material online). Interestingly, the orientation of the bias is different whether the G + C changing position is the third (i.e., the most synonymous) or not. Hence, different combinations of position specific G + C changes may result in different types of biases.

**Fig. 2.**
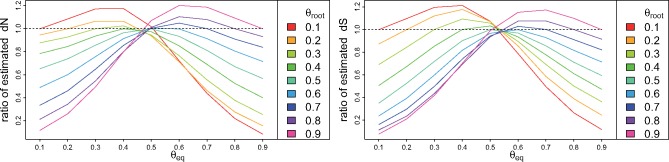
Ratio of substitution rates estimated with stationary model over substitution rates estimated with nonstationary model. Sequences were simulated with changing G + C content and ω=0.1. Left: d*N*. Right: d*S*. *θ*_root_: G + C frequency in the root sequence. *θ*_eq_: G + C equilibrium frequency of the simulation model.

As far as (non)synonymous substitution rates are concerned, assuming stationarity biases both the estimates of d*N* and d*S* in similar ways (fig. 1*a* and *c*). These values are mostly underestimated when equilibrium GC is very different from 0.5 and GC content changes (either up or down) (fig. 2). Thus in these cases the inferred trees are too short.

To check that these biases are not due to our method, we also performed the same estimates under stationary assumption with *codeml* (Yang and Nielsen 2000), and the results exhibit similar biases (see supplementary fig. S9, Supplementary Material online).

All these biases are corrected when using our approach with a nonstationary model, both for *ω* (supplementary fig. S1*b*, Supplementary Material online), d*N*, d*S*, and $\frac{dN}{dS}$ (fig. 1*b*, *d*, and *f*), and even when the nonstationarity differs among codon positions (see supplementary figs. S6–S8, Supplementary Material online).

Interestingly, we observed that estimates of d*N* and d*S* decrease with equilibrium GC content (fig. 1*b* and *d*). This is not due to our method, since on stationary processes, estimates of d*N* and d*S* computed with codeml show a similar trend (see the dashed line in supplementary fig. S9, Supplementary Material online). This relationship between d*N* or d*S* and equilibrium GC content depends on the value of omega. When *ω* is low, this correlation is negative (fig. 1*b* and *d*), when *ω* equals 1 the correlation is null (supplementary fig. S3, Supplementary Material online), and it gets positive as *ω* gets higher than 1 (e.g., for *ω* = 2 see supplementary fig. S5, Supplementary Material online).

**Fig. 3.**
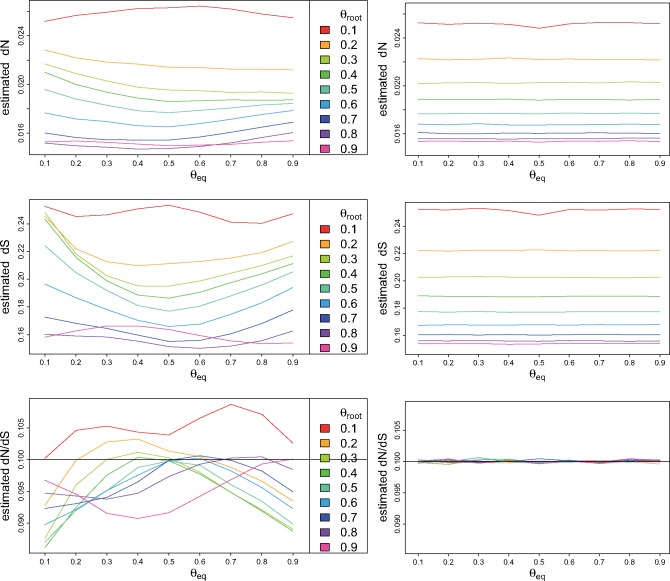
Estimate of d*N*, d*S*, and $\frac{dN}{dS}$ on primate branches with a stationary model (left), and a nonstationary nonhomogeneous model (right), on simulated data with changing G + C content on dog and rodent branches, and ω=0.1. *θ*_root_: G + C frequency in the root and primates sequences. *θ*_eq_: G + C equilibrium frequency of the simulation model on dog and rodent branches.

It should be noted that when the dynamics of GC content is heterogeneous, the bias is not systematically in the same direction whether GC increases (or decreases), but depends also on the GC of other branches, since a stationary modeling (hence homogeneous) will estimate its GC equilibrium from all branches. For example, on the same tree, we took into consideration a model with stationary GC from the root to the primate leaves, and changing GC on the branches leading to dog and to rodents. As shown in figure 3, estimates of $\frac{dN}{dS}$ on primate branches are biased with the hypothesis of stationarity, even though the process is indeed stationary on these branches. But the nonstationarity on the other branches misleads the estimated stationary model.

### Study on Mammalian Data Set

We performed two different maximum likelihood estimates of the mammalian data set: a stationary homogeneous YN98 + F3X4 model (21 branch and model parameters), and a nonstationary nonhomogeneous model (31 additional parameters) with three homogeneous YN98 models, one for the primate clade, one for the rodent clade and one for the dog branch. We used three models to match the heterogeneity in equilibrium GC content found between these clades Romiguier *et al.* (2010). We computed d*N* (resp. d*S*) in the primate clade by summing the stochastic mapping d*N* (resp. d*S*) of all branches of this clade.

**Fig. 4.**
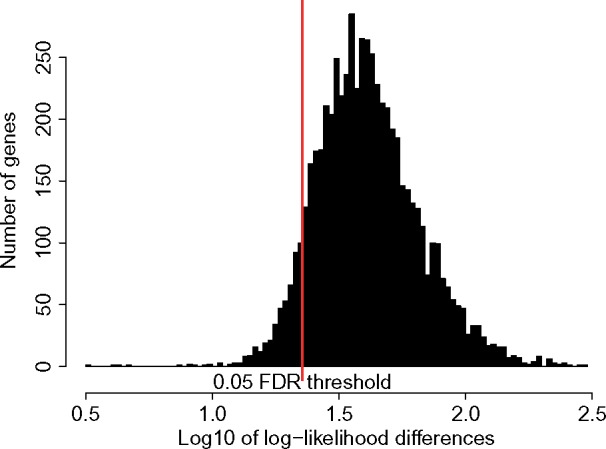
Log10 of the differences in log-likelihoods between stationary and nonstationary models on mammalian data. The red line stands for the 5% FDR threshold.

Since the models are nested, we performed likelihood ratio tests on all estimates, and corrected multiple testing using Benjamini–Hochberg correction. The increase in likelihood is significant (using an LRT test with 31 degrees of freedom) with a 1% FDR value, in 83.4% of the genes (fig. 4).

**Fig. 5.**
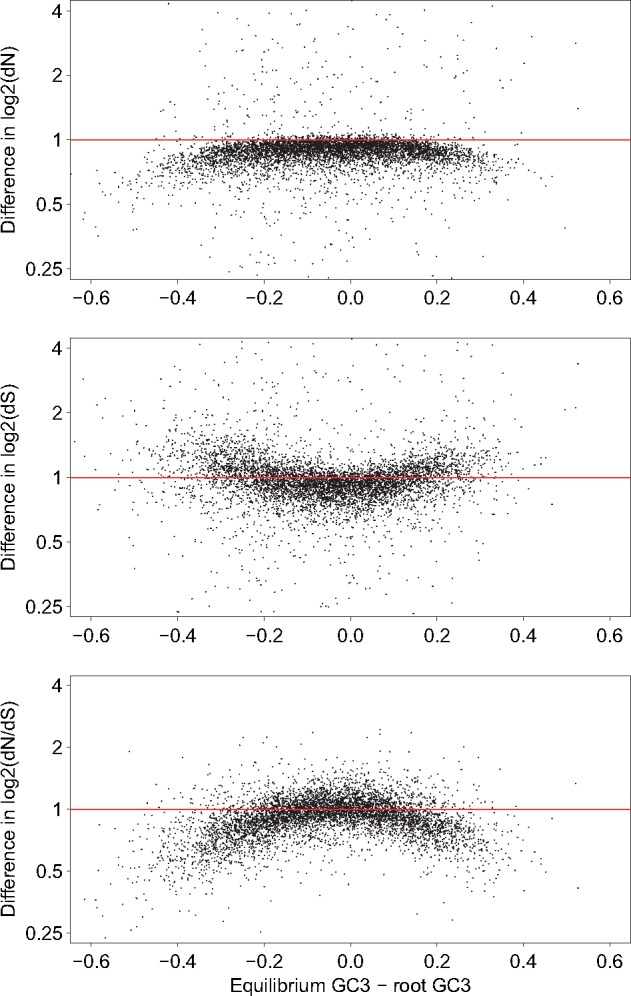
log2 of the ratios of estimates of d*N*, d*S*, and d*N*/d*S* with a stationary model over the estimates with a nonstationary model, according to the change in *GC*3 content in the primate clade.

**Fig. 6.**
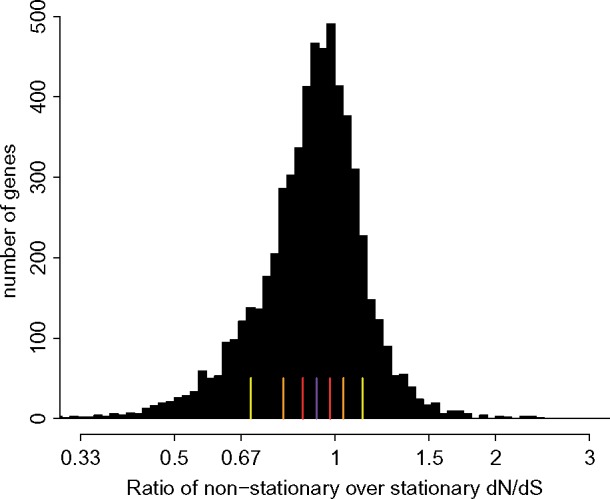
Histogram of the ratios in estimates of *ω* in stationary model over nonstationary model on mammalian data. Yellow, orange, and red lines stand for 12.5%, 25%, and 37.5% quantiles. The purple line represents the median.

If we compare the estimates of stationary versus nonstationary modeling, we see that the estimates of d*N* are mostly lower, but not correlated with the evolution of GC-content at the third codon position (GC3) (fig. 5). On the contrary, we see an influence of the evolution in GC3 on the bias in the estimate of d*S*, and then a more important underestimate of $\frac{dN}{dS}$ with genes far from stationarity in GC3. As noticed in the simulation section, the bias is not correlated with the sign of change in GC3 because we performed a nonhomogeneous modeling, and the bias depends also on the evolution of GC content in the other branches. However, the effect is quite noticeable: the relative error on *ω* estimate is at least 10% for 59% of the genes, or at least 33% for 13.4% of the genes (fig. 6).

## Discussion

Our analyses, both on simulated and empirical data sets, show that estimates of d*N*, d*S*, and *ω* can be biased when using standard methods, which assume sequence stationarity. The strength of the bias depends on the gap between the equilibrium and the actual base composition. Generally, estimates of *ω* more strongly withstand this bias than those of d*N* or d*S* (fig. 1), but in extreme cases our simulations showed a 2-fold difference between true and estimated value of *ω*. This bias can have a profound impact for analyses aimed at comparing average values of *ω* among large gene sets. For instance, to investigate the parameters that explain variations in the efficacy of selection, many studies have compared the genome-wide average of *ω* across different taxa (e.g., Galtier and Schierup 2016). The genome-wide average of *ω* varies from 0.13 to 0.17 among 48 bird species (Weber et al. 2014), and from 0.10 to 0.22 among 106 amniote species (Figuet et al. 2016). Thus, at this scale, systematic errors in the estimate of *ω* caused by differences in the equilibrium base composition along lineages might have an important impact on observed patterns.

The method we developed, based on stochastic substitution mapping, provides unbiased estimates of d*N*, d*S*, and $\frac{dN}{dS}$. Moreover, this method can be used with any type of codon modeling, as long as neutrality (i.e., absence of selection) is possible in this modeling through specific parameter values (such as *ω* = 1 for the YN98 model). As these estimates maximize the expected likelihood, given a model, they can be used in an Expectation-Maximization procedure, to optimize branch specific estimate of selection.

The classical approach to detect episodic selection regimes consists in estimating *ω* on specific branches (or set of branches) (e.g., for episodic positive selection; Messier and Stewart 1997; Kosakovsky Pond and Frost 2005). One difficulty is that estimating *ω* by maximum likelihood on each branch (or set of branches) may entail convergence problems (especially with large trees), and is quite computer-intensive (Romiguier et al. 2012). Thus, the standard practice consists in assuming a homogeneous model on the tree in a maximum likelihood first step, and in a second step to look for heterogeneity. With this approach, exploring variation of *ω* along the tree without any a priori can be quite tedious. The substitution mapping approach provides countings that allow either branch specific analysis (Lemey et al. 2012), or recursive splitting of the tree in homogeneous subtrees (Dutheil et al. 2012). *mapdNdS* directly provides d*N* and d*S* on each branch of the tree. Consequently, by summing the d*N* (and d*S*) over a given set of branches, it is possible to estimate the overall (non)synonymous rate of substitution along these branches, and thereby to infer the corresponding $\frac{dN}{dS}$ ratio. Thus, one important practical interest of this approach is that it is straightforward to estimate selection on any subset of the tree to search for signals of episodic selection regimes.

It seems also straightforward to adapt this approach in the estimate of selection on specific sites, and indeed it is already possible with “simple” models such as the ones we took into consideration in this article. However, most site-specific studies use site-models to model the heterogeneity in selection along the sequence (Yang et al. 2000). Results of substitution mapping depend on the model used, and it seems reasonable to use similar site-models in the case of heterogeneous sequences.

As described by Minin and Suchard (2008), in addition to computing the expectation of counts and times on branches, it is possible to compute their variance (and other moments). This would provide statistical information on the accuracy of the estimates of the denominators and numerators of d*N* and d*S*. We plan to implement the algorithm proposed by Dhar and Minin (2017) to study how this variance can help to measure confidence in the estimates of substitution rates. Providing confidence intervals would be particularly useful for site-specific or branch-specific rates, which are expected to be noisier, given that they are estimated from more limited data.

## Materials and Methods

### Stochastic Mapping

The aim of this approach is to compute d*N* and d*S* for a set of sequences along a phylogenetic tree with stochastic mapping. More formally, given a set of sequences *D* and a phylogenetic tree *T*, each sequence of *D* is the result of a substitution process from a root sequence along the branches of *T*. This substitution process is assumed to follow a continuous time Markovian model M, with generator Q, and starting distribution R (if the process is stationary R is the equilibrium distribution of M). We denote by L a set of substitutions considered, that is, synonymous or nonsynonymous substitutions.

Several substitution mapping methods make it possible to compute $E(N_{L}|b,D,M)$, the expectation on all scenarios of the number $N_{L}$ of L− events that occur on this process on a branch *b* given data *D* (Tataru and Hobolth 2011). Those computations rely on the joint a posteriori probabilities of the states at the tips of *b*, which are defined by model M whether it is stationary or not.

Next, we define $A_{L}^{M\prime }$ as the ability of a model M′ to perform substitutions in L (see Supplementary Material online for a more formal definition). In a short time dτ, from a starting state *s*, $A_{L}^{M\prime }d\tau$ is the sum of the rates of the substitutions that start from *s* and belong to L (for example, the sum of the rates of synonymous substitutions from codon AAA). On a branch *b*, we compute the mean value of this instantaneous definition all along the branch, and we take into consideration the expectation of this over all scenarios given data *D* and model M: $E(A_{L}^{M\prime }|b,D,M)$. We show that this expectation can be computed efficiently using the stochastic mapping approach (see Supplementary Material online).

In theory M should be the true model, but in practice will be the most likely model. Please note that the model used to define the ability $A_{L}^{M\prime }$ can be different from M.

How can we use these definitions to compute relevant d*N* and d*S*? First we set up the normalization of the counts (aka the “per [non]synonymous site” feature) properly. At each time, the property of a site to be (non)synonymous is based on the rates of all the (non)synonymous substitutions this site can undergo. These rates depend on the model under consideration. For example, a site with the codon AAA (for which the only synonymous codon is AAG) will be more synonymous with a model that favors A and G nucleotides than with a model that favors C and T. So the normalization must be defined through a model similar to M, but defined as neutral, that is, which does not favor synonymous or nonsynonymous substitutions in its definition (Yang and Nielsen 2000; Kosakovsky Pond and Frost 2005). We denote by $M^{0}$ this neutral version of M, and by $A_{m}^{0}bL$ the ability of this neutral model to perform L-events. In the case of model YN98 (Yang and Nielsen 1998), $M^{0}$ is built as M, with *ω* = 1.

Hence, to compute relevant d*N* and d*S* along a branch *b*, the expected counts of (non)synonymous substitutions will be normalized by the expected mean along the branch of potential substitutions according to the same model but without selection, which is by definition the ability of the neutral model $M^{0}$.

The ratio $\frac{E(N_{L}|b,D,M)}{E(A_{L}^{0}|b,D,M)}$ is then considered an a posteriori normalized count of the L− events on branch *b*. Since the models are built on codon sequences, they are normalized such that there is one substitution per codon per unit of time on sequences at equilibrium. It is then straightforward to see that the ability of a model to perform any substitution equals 1 per unit of time per codon. In the usual definitions of d*N* and d*S*, the normalization is not “per codon” but “per nucleotide,” which means the ability of a model to perform any substitution should be 1 per unit of time per nucleotide, that is, three times the previous one. Finally, we obtain the equivalents of d*N* and d*S* in the methodology of stochastic mapping: $\frac{E(N_{L}|b,D,M)}{3E(A_{L}^{0}|b,D,M)}$.

We show in the Supplementary Material online that these estimates maximize on each branch the expected likelihood of the data, given M and *T*. These estimates correspond then to the first step of an Expectation-Maximization procedure (as in Holmes and Rubin 2002).

### Applications

To investigate the bias induced by stationarity assumption, we use stochastic mapping to compute relevant d*N*, d*S*, and $\frac{dN}{dS}$ estimates on simulated and empirical sequence data sets, both of which are subject to changes in GC content. The same model has been used, aka the model proposed by Yang and Nielsen in 1998 (Yang and Nielsen 1998) (denoted by “YN98”), both in homogeneous and nonhomogeneous (or branch) modeling. To model a nonstationary process, root codon frequencies must be introduced, and then the frequencies evolve continuously from this root distribution towards the equilibrium distribution(s) of the model(s). To reduce the number of parameters to estimate, root and equilibrium codon frequencies are computed as products of position nucleotide frequencies instead of a full parametrization of the codon frequencies (61 parameters). In simulations, when G + C content evolution is not position-specific, nucleotide frequencies are modeled as identical for all positions (denoted by “F1X4,” with 2 × 3 parameters), and when G + C content evolution is position-specific nucleotide frequencies are position-specific inside codons (denoted “F3X4,” with 2 × 9 parameters, because of three equilibrium frequencies). For real data set analyses, F3X4 modeling is used. In both cases, codon frequencies are normalized such that stop codon frequencies are set to 0.

In a first step, parameter *ω* is estimated through maximum likelihood computation of model, root frequencies and branch lengths on each alignment. Then, in a second step, d*N* and d*S* are computed using normalized stochastic mapping from this optimized model and tree.

This procedure, called *mapdNdS*, has been implemented in the Bio ++ program suite (Guéguen et al. 2013). It can thus easily be used on the numerous models that are available in this suite, and most importantly in any nonhomogeneous modeling. Moreover, it can produce both site-specific and/or branch-specific estimates.

This suite was used for simulations, maximum likelihood estimates and stochastic mapping computations.

### Data

#### Simulated Data Set

**Fig. 7.**
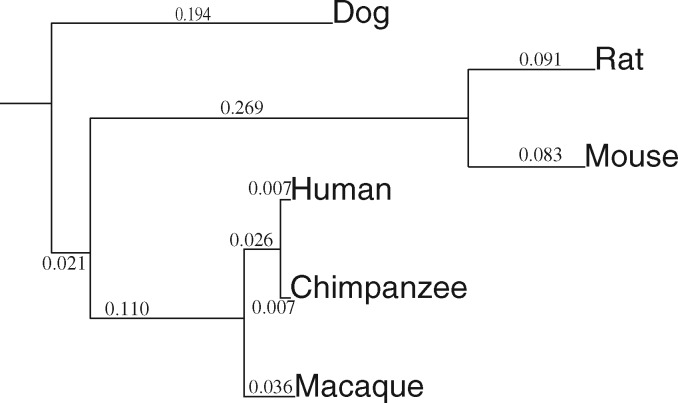
Phylogeny of the studied species in the mammalian data set. The same tree is used for simulation with two different theta values: *θ*_root_ is the G + C probability at the root, *θ*_eq_ is the equilibrium G + C probability.

To study the influence of the nonstationarity in G + C content on the maximum likelihood estimate of *ω*, we simulated the evolution of 100 coding sequences of 3000 codons. Each simulation started from an ancestral sequence with a determined proportion of G + C, noted *θ*_root,_ and ran along the tree depicted in figure 7, using a homogeneous YN98 + F1X4 model with a determined G + C equilibrium frequency, noted *θ*_eq._ Each *θ* value (*θ*_eq_ and *θ*_root)_ ranged from 0.1 to 0.9 per step of 0.1. We simulated negative, weakly negative, neutral, and weakly positive selection (resp. ω=0.1, ω=0.9, *ω* = 1, ω=1.1).

#### Mammalian Data Set

From the data studied (Kosiol et al. 2008), we retrieved 6,055 sequence alignments of orthologous genes present in human, chimpanzee, macaque, mouse, rat, and dog genomes.

## Supplementary Material

Supplementary data are available at *Molecular Biology and Evolution* online.

## Availability

*mapdNdS* has been implemented in the Bio++ suite (Guéguen et al. 2013). The maximum likelihood program is called bppml, and is available at the address http://bioweb.me/bppsuite. The stochastic mapping program is called mapnh, and is available at the address http://bioweb.me/testnh.

A short tutorial about model inference and stochastic mapping as described in this article is available here: http://bioweb.me/mapdNdS.

## Supplementary Material

Supplementary Data

## References

[msx308-B1] Bernardi G, Olofsson B, Filipski J, Zerial M, Salinas J, Cuny G, Meunier-Rotival M, Rodier F. 1985 The mosaic genome of warm-blooded vertebrates. Science 228(4702): 953–958. 4001930 10.1126/science.4001930

[msx308-B2] Bolivar P, Mugal CF, Nater A, Ellegren H. 2016 Recombination rate variation modulates gene sequence evolution mainly via GC-biased gene conversion, not Hill–Robertson interference, in an avian system. Mol Biol Evol. 33(1): 216–227.http://dx.doi.org/10.1093/molbev/msv214 26446902 10.1093/molbev/msv214 PMC4693978

[msx308-B3] Dhar A, Minin VN. 2017 Calculating higher-order moments of phylogenetic stochastic mapping summaries in linear time. J Comput Biol. 24(5): 377–399.http://dx.doi.org/10.1089/cmb.2016.0172 28177780 10.1089/cmb.2016.0172 PMC5470758

[msx308-B4] Dufresne A, Salanoubat M, Partensky F, Artiguenave F, Axmann IM, Barbe V, Duprat S, Galperin MY, Koonin EV, Gall FL, 2003 Genome sequence of the cyanobacterium *Prochlorococcus marinus* SS120, a nearly minimal oxyphototrophic genome. Proc Natl Acad Sci U S A. 100(17): 10020–10025. 12917486 10.1073/pnas.1733211100 PMC187748

[msx308-B5] Dufresne A, Garczarek L, Partensky F. 2005 Accelerated evolution associated with genome reduction in a free-living prokaryote. Genome Biol. 6(2): 1–10. 10.1186/gb-2005-6-2-r14 PMC551534 15693943

[msx308-B6] Duret L, Arndt PF. 2008 The impact of recombination on nucleotide substitutions in the human genome. PLoS Genet. 4(5): e1000071. 18464896 10.1371/journal.pgen.1000071 PMC2346554

[msx308-B7] Dutheil J, Pupko T, Jean-Marie A, Galtier N. 2005 A model-based approach for detecting coevolving positions in a molecule. Mol Biol Evol. 22(9): 1919–1928.http://dx.doi.org/10.1093/molbev/msi183 15944445 10.1093/molbev/msi183

[msx308-B8] Dutheil JY, Galtier N, Romiguier J, Douzery EJ, Ranwez V, Boussau B. 2012 Efficient selection of branch-specific models of sequence evolution. Mol Biol Evol. 29(7): 1861–1874. 22319139 10.1093/molbev/mss059

[msx308-B9] Figuet E, Nabholz B, Bonneau M, Mas Carrio E, Nadachowska-Brzyska K, Ellegren H, Galtier N. 2016 Life history traits, protein evolution, and the nearly neutral theory in amniotes. Mol Biol Evol. 33(6): 1517–1527. 26944704 10.1093/molbev/msw033

[msx308-B10] Galtier N, Schierup MH. 2016 Adaptive protein evolution in animals and the effective population size hypothesis. PLoS Genet. 12(1): e1005774. 26752180 10.1371/journal.pgen.1005774 PMC4709115

[msx308-B11] Galtier N, Duret L, Glemin S, Ranwez V. 2009 GC-biased gene conversion promotes the fixation of deleterious amino acid changes in primates. Trends Genet. 25(1): 1–5.http://dx.doi.org/10.1016/j.tig.2008.10.011 19027980 10.1016/j.tig.2008.10.011

[msx308-B12] Goldman N, Yang Z. 1994 A codon-based model of nucleotide substitution for protein-coding DNA sequences. Mol Biol Evol. 11(5): 725–736. 7968486 10.1093/oxfordjournals.molbev.a040153

[msx308-B13] Guéguen L, Gaillard S, Boussau B, Gouy M, Groussin M, Rochette N, Bigot T, Fournier D, Pouyet F, Cahais V, 2013 Bio ++: efficient extensible libraries and tools for computational molecular evolution. Mol Biol Evol. 30(8): 1745–1750. 23699471 10.1093/molbev/mst097

[msx308-B14] Guindon S, Rodrigo AG, Dyer KA, Huelsenbeck JP. 2004 Modeling the site-specific variation of selection patterns along lineages. Proc Natl Acad Sci U S A. 101(35): 12957–12962. 15326304 10.1073/pnas.0402177101 PMC516501

[msx308-B15] Hobolth A, Stone E. 2009 Simulation from endpoint-conditioned, continuous-time Markov chains on a finite state space, with applications to molecular evolution. Ann Appl Stat. 3(3): 1204 http://dx.doi.org/10.1214/09-AOAS247 20148133 10.1214/09-AOAS247 PMC2818752

[msx308-B16] Holmes I, Rubin G. 2002 An expectation maximization algorithm for training hidden substitution models. J Mol Biol. 317(5): 753–764. 11955022 10.1006/jmbi.2002.5405

[msx308-B17] Itoh T, Martin W, Nei M. 2002 Acceleration of genomic evolution caused by enhanced mutation rate in endocellular symbionts. Proc Natl Acad Sci U S A. 99(20): 12944–12948.http://dx.doi.org/10.1073/pnas.192449699 12235368 10.1073/pnas.192449699 PMC130565

[msx308-B18] Kosakovsky Pond SL, Frost SD. 2005 Not so different after all: a comparison of methods for detecting amino acid sites under selection. Mol Biol Evol. 22(5): 1208–1222.http://dx.doi.org/10.1093/molbev/msi105 15703242 10.1093/molbev/msi105

[msx308-B19] Pond SLK, Frost SDW, Muse SV. 2005 HyPhy: hypothesis testing using phylogenies. Bioinformatics 21(5): 676–679.http://dx.doi.org/10.1093/bioinformatics/bti079 15509596 10.1093/bioinformatics/bti079

[msx308-B20] Kosiol C, Vinar T, da Fonseca RR, Hubisz MJ, Bustamante CD, Nielsen R, Siepel A. 2008 Patterns of positive selection in six mammalian genomes. PLoS Genet. 4(8): e1000144. 18670650 10.1371/journal.pgen.1000144 PMC2483296

[msx308-B21] Kumar S. 2005 Molecular clocks: four decades of evolution. Nat Rev Genet. 6(8): 654–662.http://dx.doi.org/10.1038/nrg1659 16136655 10.1038/nrg1659

[msx308-B22] Kumar S, Subramanian S. 2002 Mutation rates in mammalian genomes. Proc Natl Acad Sci U S A. 99(2): 803–808.http://dx.doi.org/10.1073/pnas.022629899 11792858 10.1073/pnas.022629899 PMC117386

[msx308-B23] Lemey P, Minin VN, Bielejec F, Kosakovsky Pond SL, Suchard MA. 2012 A counting renaissance: combining stochastic mapping and empirical Bayes to quickly detect amino acid sites under positive selection. Bioinformatics 28(24): 3248–3256.http://dx.doi.org/10.1093/bioinformatics/bts580 23064000 10.1093/bioinformatics/bts580 PMC3579240

[msx308-B24] Li WH, Wu CI, Luo CC. 1985 A new method for estimating synonymous and nonsynonymous rates of nucleotide substitution considering the relative likelihood of nucleotide and codon changes. Mol Biol Evol. 2(2): 150–174. 3916709 10.1093/oxfordjournals.molbev.a040343

[msx308-B25] Messier W, Stewart CB. 1997 Episodic adaptive evolution of primate lysozymes. Nature 385(6612): 151–154.http://dx.doi.org/10.1038/385151a0 8990116 10.1038/385151a0

[msx308-B26] Minin V, Suchard M. 2008 Fast, accurate and simulation-free stochastic mapping. Philos Trans Roy Soc B. 363(1512): 3985–3995. 10.1098/rstb.2008.0176 PMC2607419 18852111

[msx308-B27] Moran NA. 1996 Accelerated evolution and Muller’s rachet in endosymbiotic bacteria. Proc Natl Acad Sci U S A. 93(7): 2873–2878. 8610134 10.1073/pnas.93.7.2873 PMC39726

[msx308-B28] Moran NA, McCutcheon JP, Nakabachi A. 2008 Genomics and evolution of heritable bacterial symbionts. Annu Rev Genet. 42(1): 165–190.http://dx.doi.org/10.1146/annurev.genet.41.110306.130119 18983256 10.1146/annurev.genet.41.110306.130119

[msx308-B29] Mouchiroud D, D'Onofrio G, Aïssani B, Macaya G, Gautier C, Bernardi G. 1991 The distribution of genes in the human genome. Gene 100:181–187. 2055469 10.1016/0378-1119(91)90364-h

[msx308-B30] Nei M, Gojobori T. 1986 Simple methods for estimating the numbers of synonymous and nonsynonymous nucleotide substitutions. Mol Biol Evol. 3(5): 418–426. 3444411 10.1093/oxfordjournals.molbev.a040410

[msx308-B31] Nielsen R. 2002 Mapping mutations on phylogenies. Syst Biol. 51(5): 729–739.http://dx.doi.org/10.1080/10635150290102393 12396587 10.1080/10635150290102393

[msx308-B32] O’Brien J, Minin V, Suchard M. 2009 Learning to count: Robust estimates for labeled distances between molecular sequences. Mol Biol Evol. 26(4): 801–814. 19131426 10.1093/molbev/msp003 PMC2734148

[msx308-B33] Paul S, Dutta A, Bag SK, Das S, Dutta C. 2010 Distinct, ecotype-specific genome and proteome signatures in the marine cyanobacteria *Prochlorococcus*. BMC Genomics 11(1): 103. http://dx.doi.org/10.1186/1471-2164-11-103 20146791 10.1186/1471-2164-11-103 PMC2836286

[msx308-B34] Prez-Brocal V, Gil R, Ramos S, Lamelas A, Postigo M, Michelena JM, Silva FJ, Moya A, Latorre A. 2006 A small microbial genome: the end of a long symbiotic relationship? Science 314(5797): 312–313. 17038625 10.1126/science.1130441

[msx308-B35] Rocap G, Larimer FW, Lamerdin J, Malfatti S, Chain P, Ahlgren NA, Arellano A, Coleman M, Hauser L, Hess WR, 2003 Genome divergence in two *Prochlorococcus* ecotypes reflects oceanic niche differentiation. Nature 424(6952): 1042–1047. 12917642 10.1038/nature01947

[msx308-B36] Romiguier J, Ranwez V, Douzery E, Galtier N. 2010 Contrasting GC-content dynamics across 33 mammalian genomes: relationship with life-history traits and chromosome sizes. Genome Res. 20(8): 1001–1009.http://dx.doi.org/10.1101/gr.104372.109 20530252 10.1101/gr.104372.109 PMC2909565

[msx308-B37] Romiguier J, Figuet E, Galtier N, Douzery EJP, Boussau B, Dutheil JY, Ranwez V, Liberles D. 2012 Fast and robust characterization of time-heterogeneous sequence evolutionary processes using substitution mapping. PLoS One 7(3): 1–10. 10.1371/journal.pone.0033852 PMC3313935 22479459

[msx308-B38] Tataru P, Hobolth A. 2011 Comparison of methods for calculating conditional expectations of sufficient statistics for continuous time Markov chains. BMC Bioinformatics 12(1): 465–475. 22142146 10.1186/1471-2105-12-465 PMC3329461

[msx308-B39] van Ham RCHJ, Kamerbeek J, Palacios C, Rausell C, Abascal F, Bastolla U, Fernández JM, Jiménez L, Postigo M, Silva FJ, 2003 Reductive genome evolution in *Buchnera aphidicola*. Proc Natl Acad Sci U S A. 100(2): 581–586., 12522265 10.1073/pnas.0235981100 PMC141039

[msx308-B40] Weber CC, Nabholz B, Romiguier J, Ellegren H. 2014 Kr/Kc but not dN/dS correlates positively with body mass in birds, raising implications for inferring lineage-specific selection. Genome Biol. 15(12): 542 http://dx.doi.org/10.1186/s13059-014-0542-8 25607475 10.1186/s13059-014-0542-8 PMC4264323

[msx308-B41] Wernegreen JJ, Moran NA. 1999 Evidence for genetic drift in endosymbionts (*Buchnera*): analyses of protein-coding genes. Mol Biol Evol. 16(1): 83–97.http://dx.doi.org/10.1093/oxfordjournals.molbev.a026040 10331254 10.1093/oxfordjournals.molbev.a026040

[msx308-B42] Wolfe KH, Sharp PM, Li WH. 1989 Mutation rates differ among regions of the mammalian genome. Nature 337(6204): 283–285.http://dx.doi.org/10.1038/337283a0 2911369 10.1038/337283a0

[msx308-B43] Yang Z. 2007 PAML 4: phylogenetic analysis by maximum likelihood. Mol Biol Evol. 24(8): 1586–1591.http://dx.doi.org/10.1093/molbev/msm088 17483113 10.1093/molbev/msm088

[msx308-B44] Yang Z, Bielawski JP. 2000 Statistical methods for detecting molecular adaptation. Trends Ecol Evol. 15(12): 496–503.http://dx.doi.org/10.1016/S0169-5347(00)01994-7 11114436 10.1016/S0169-5347(00)01994-7 PMC7134603

[msx308-B45] Yang Z, Nielsen R. 1998 Synonymous and nonsynonymous rate variation in nuclear genes of mammals. J Mol Evol. 46(4): 409–418.http://dx.doi.org/10.1007/PL00006320 9541535 10.1007/pl00006320

[msx308-B46] Yang Z, Nielsen R. 2000 Estimating synonymous and nonsynonymous substitution rates under realistic evolutionary models. Mol Biol Evol. 17(1): 32–43.http://dx.doi.org/10.1093/oxfordjournals.molbev.a026236 10666704 10.1093/oxfordjournals.molbev.a026236

[msx308-B47] Yang Z, Nielsen R, Goldman N, Pedersen AM. 2000 Codon-substitution models for heterogeneous selection pressure at amino acid sites. Genetics 155(1): 431–449. 10790415 10.1093/genetics/155.1.431 PMC1461088

[msx308-B48] Yu T, Li J, Yang Y, Qi L, Chen B, Zhao F, Bao Q, Wu J. 2012 Codon usage patterns and adaptive evolution of marine unicellular cyanobacteria *Synechococcus* and *Prochlorococcus*. Mol Phylogenet Evol. 62(1): 206–213.http://dx.doi.org/10.1016/j.ympev.2011.09.013 22040764 10.1016/j.ympev.2011.09.013

