# Supplementary material for: Unbiased Estimate of Synonymous and Nonsynonymous Substitution Rates with Nonstationary Base Composition
Source: Mol Biol Evol. 2017 Dec 6;35(3):734–42. doi: 10.1093/molbev/msx308 (PMC5850866; doi:10.1093/molbev/msx308)
Supplement: Supplementary Data [file msx308_supp.pdf]

Unbiased estimate of synonymous and non-synonymous substitution rates with non-stationary base composition: supplementary material

Laurent Guéguen and Laurent Duret

1 Mathematics

1.1 Ability

Here we describe how stochastic mapping is used to compute the ability of a model for a set of sequences along a phylogenetic tree.

We denote by D a set of sequences in an alphabet $\mathcal{A}$, and by T a phylogenetic tree. On each site, each sequence of D is the result of a substitution process from a root sequence along the branches of T. On a given branch b of length t, this substitution process can be represented by a continuous time Markov process $(X(\tau))_\tau$.

We define $\mathbb{E} = \{(a,' a) \in \mathcal{A} \times \mathcal{A}; a \neq a'\}$ the set of all substitutions, and focus on a subset $\mathbb{L}$ of events ($\mathbb{L} \subset \mathbb{E}$). These events are named $\mathbb{L}-$events. In our case, the $\mathbb{L}-$events are the synonymous substitutions or the non-synonymous substitutions and $\mathcal{A}$ is the set of codons.

$N_\mathbb{L}$ denotes the number of $\mathbb{L}-$events that occur along process X. Since X is unknown, $N_\mathbb{L}$ is unknown. Substitution mapping approach is used to compute the expectation of $N_\mathbb{L}$ over the distribution of X, given branch b, model $\mathcal{M}$, and data D, *i.e.* $E(N_\mathbb{L}|b, \mathcal{M}, D)$.

Now we define and compute the ability of a model $\mathcal{M}'$ (with generator $\mathcal{Q}'$) along this process X.

At time τ, during a short time $d\tau$, we define the instantaneous **ability** of $\mathcal{M}'$ to perform $\mathbb{L}-$event, $A_\mathbb{L}^{\mathcal{Q}'}(\tau)$, as the expectation - on $X(\tau)$ - of the number of $\mathbb{L}-$events that would have been performed by a process following model $\mathcal{M}'$ during $d\tau$:

$$
\begin{aligned}
A_\mathbb{L}^{\mathcal{Q}'}(\tau) &= \sum_{(a,a') \in \mathbb{L}} \mathcal{Q}'_{a,a'}.P(X(\tau) = a)d\tau \\
&= \sum_{a \in \mathcal{A}} \mathcal{Q}'_{a,\mathbb{L}} P(X(\tau) = a)d\tau
\end{aligned}
$$

where $\mathcal{Q}'_{a,\mathbb{L}} = \sum_{a' \in \mathcal{A};(a,a') \in \mathbb{L}} \mathcal{Q}'_{a,a'}$.

The ability $A_\mathbb{L}^{\mathcal{Q}'}$ is the mean value of this sum along the process X:

$$
\begin{aligned}
A_{\mathbb{L}}^{\mathcal{Q}'} &= \frac{1}{t}\int_{\tau=0}^{t} A_{\mathbb{L}}^{\mathcal{Q}'}(\tau)d\tau \\
&= \frac{1}{t}\int_{\tau=0}^{t}\sum_{a\in\mathcal{A}} \mathcal{Q}'_{a,\mathbb{L}} P(X(\tau)=a)d\tau = \frac{1}{t}\sum_{a\in\mathcal{A}} \mathcal{Q}'_{a,\mathbb{L}}\int_{\tau=0}^{t} P(X(\tau)=a)d\tau \\
&= \frac{1}{t}\sum_{a\in\mathcal{A}} \mathcal{Q}'_{a,\mathbb{L}}\mathcal{T}_a
\end{aligned}
$$

where $\mathcal{T}_a = \int_{\tau=0}^{t} P(X(\tau)=a)d\tau$ is the time spent by X in state a.

As for stochastic mapping, the expectation of $A_{\mathbb{L}}^{\mathcal{Q}'}$ over all X, $E(A_{\mathbb{L}}^{\mathcal{Q}'}|b,M,D)$ can be efficiently computed. In Minin and Suchard (2008), $t.A_{\mathbb{L}}^{\mathcal{Q}'}$ is defined as the **reward** of vector $\mathcal{Q}'_{\mathbb{L}} = (\mathcal{Q}'_{a,\mathbb{L}})_a$, which expectation over all scenarios given branch b, model $\mathcal{M}$, and data D, can be computed in the same way as $E(N_{\mathbb{L}}|b,M,D)$.

1.2 Estimates of dN and dS

Here, we show that the proposed estimates of dN and dS, using the ability of a neutral model, are the most likely on a branch b of a tree T, given a model M and data D.

Given a model M' with generator Q', the log-likelihood of a process X along a branch b is:

$$
lL(X|M') = \sum_{a\in\mathcal{A}} Q'_{aa}.\mathcal{T}_a + \sum_{(a,a')\in\mathbb{E}} N_{aa'}\log(Q'_{aa'})
$$

where $\mathcal{T}_a$ is the time spent by X in state a and $N_{aa'}$ is the number of substitutions from a to a' that occurred in X on branch b. And we consider the expectation on the distribution of X given T, M and D (we remove expectation condition $|b,M,D$ for sake of clarity):

$$
\begin{aligned}
E(lL|M') &= \textstyle\sum_{a\in\mathcal{A}} E(\mathcal{T}_a).Q'_{aa} + \sum_{(a,a')\in\mathbb{E}} E(N_{aa'})\log(Q'_{aa'}) \\
&= -\textstyle\sum_{(a,a')\in\mathbb{E}} E(\mathcal{T}_a).Q'_{aa'} + \sum_{(a,a')\in\mathbb{E}} E(N_{aa'})\log(Q'_{aa'})
\end{aligned}
$$

Now, we look for model M' that maximizes this likelihood. Actually, we only focus on the factors that define non-neutrality, *i.e.* the factors that discriminate synonymous substitutions from non-synonymous substitutions. We take into consideration two sets of substitutions : $\mathbb{S}$ (resp. $\mathbb{N}$) the set of synonymous (resp. non-synonymous) substitutions. $\mathbb{S}\cup\mathbb{N}=\mathbb{E}$.

And we consider that Q' can be written as :

$$
Q'_{aa'} = \begin{cases} \alpha Q'^{0}_{aa'} & \text{if } (a,a')\in\mathbb{S} \\ \beta Q'^{0}_{aa'} & \text{if } (a,a')\in\mathbb{N} \end{cases}
$$

where $Q'^{0}_{aa'}$ does not depend on the synonymous property of the substitution from a to a' (it is the "neutral" part of Q', a part that we do not want to estimate).

Then:

$$
\begin{aligned}
E(lL|M') &= -\sum_{(a,a')\in\mathbb{S}} E(\mathcal{T}_a)\alpha Q'^0_{aa'} - \sum_{(a,a')\in\mathbb{N}} E(\mathcal{T}_a)\beta Q'^0_{aa'} \\
&\quad + \sum_{(a,a')\in\mathbb{S}} E(N_{aa'})\log(\alpha Q'^0_{aa'}) + \sum_{(a,a')\in\mathbb{N}} E(N_{aa'})\log(\beta Q'^0_{aa'}) \\
&= -\alpha t.E(A^0_{\mathbb{S}}) - \beta t.E(A^0_{\mathbb{N}}) \\
&\quad + \log(\alpha).E(N_{\mathbb{S}}) + \log(\beta).E(N_{\mathbb{N}}) + \sum_{(a,a')\mathbb{E}} E(N_{aa'})\log(Q'^0_{aa'})
\end{aligned}
$$

with $A^0 := A^{Q'^0}$.
Now, we look for which values of α and β $E(lL|M')$ is maximized:

$$
\frac{\partial E(lL|M')}{\partial \alpha} = -t.E(A^0_{\mathbb{S}}) + \frac{E(N_{\mathbb{S}})}{\alpha} = 0 \Leftrightarrow \alpha = \frac{E(N_{\mathbb{S}})}{t.E(A^0_{\mathbb{S}})}
$$

$$
\frac{\partial E(lL|M')}{\partial \beta} = -t.E(A^0_{\mathbb{N}}) + \frac{E(N_{\mathbb{N}})}{\beta} = 0 \Leftrightarrow \beta = \frac{E(N_{\mathbb{N}})}{t.E(A^0_{\mathbb{N}})}
$$

Finally, given the fixed neutral part Q'^0 (and given $T, \mathcal{M}, D$), the most-likely model on branch b is:

$$
Q'_{aa'} = \begin{cases} \frac{E(N_{\mathbb{S}})}{t.E(A^0_{\mathbb{S}})} Q'^0_{aa'} & \text{if } (a,a') \in \mathbb{S} \\ \frac{E(N_{\mathbb{N}})}{t.E(A^0_{\mathbb{N}})} Q'^0_{aa'} & \text{if } (a,a') \in \mathbb{N} \end{cases}
$$

and the most likely estimator of ω is $\frac{E(N_{\mathbb{N}})}{E(A^0_{\mathbb{N}})} \frac{E(A^0_{\mathbb{S}})}{E(N_{\mathbb{S}})}$.

dN and dS are usually defined as the (non-)synonymous numbers of substitutions **per (non-)synonymous nucleotide**. In order to fit with this definition, since Q' is normalized to perform one substitution **per codon** and **per unit of time** on a sequence at equilibrium, the computed estimates have to be divided per 3 and multiplied by the length of the branch.

References

Minin, V. and Suchard, M. 2008. Fast, accurate and simulation-free stochastic mapping. *Phil. Trans. Roy. Soc. B*, 363: 3985–3995.

2 Figures

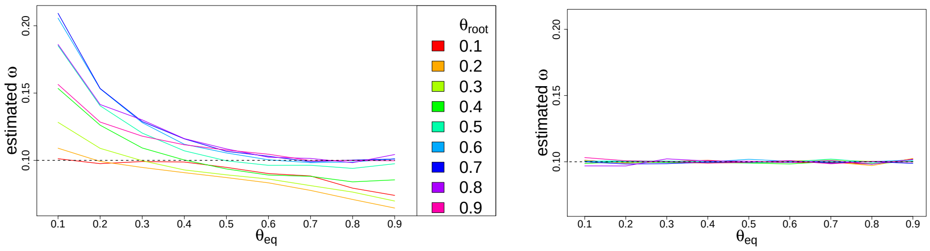

(a) (b)

Figure S1: **Estimates of** $\omega = 0.1$ with (a) a stationary model and (b) a non-stationary model, on simulated data with changing G+C content. θ_{root}: G+C frequency in the root sequence. θ_{eq}: G+C equilibrium frequency of the simulation model.

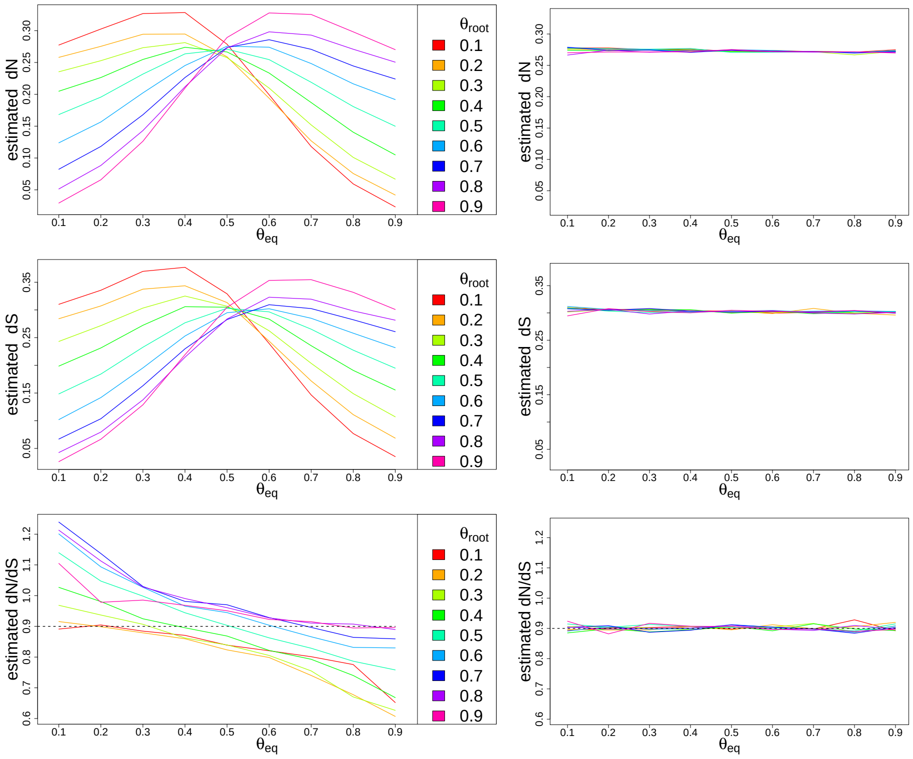

Figure S2: **Estimates of dN, dS and $\frac{dN}{dS}$** with a stationary model (left) and non-stationary model (right), on simulated data with changing G+C content and $\omega = 0.9$. θ_{root}: G+C frequency in the root sequence. θ_{eq}: G+C equilibrium frequency of the simulation model.

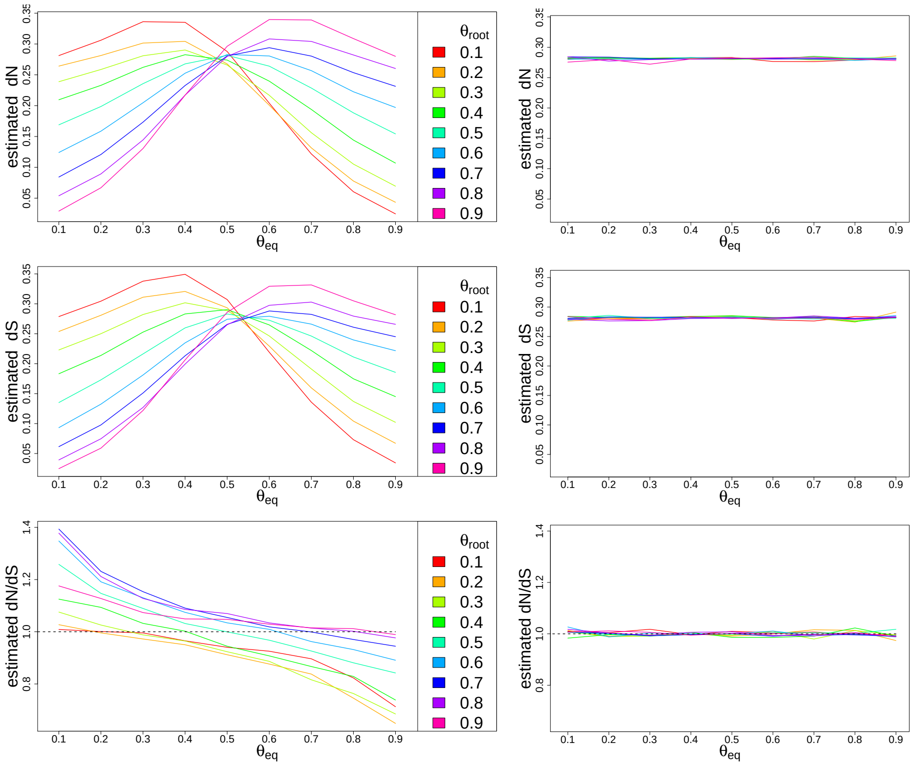

Figure S3: **Estimates of dN, dS and $\frac{dN}{dS}$** with a stationary model (left) and non-stationary model (right), on simulated data with changing G+C content and $\omega = 1$. θ_{root}: G+C frequency in the root sequence. θ_{eq}: G+C equilibrium frequency of the simulation model.

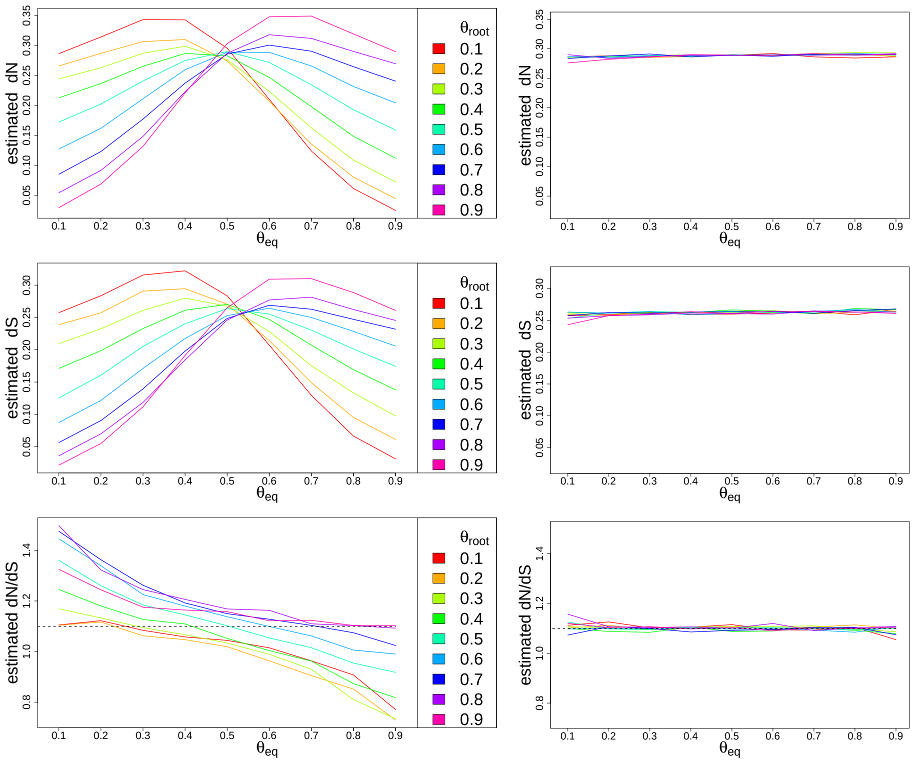

Figure S4: **Estimates of dN, dS and $\frac{dN}{dS}$** with a stationary model (left) and non-stationary model (right), on simulated data with changing G+C content and $\omega = 1.1$. θ_{root}: G+C frequency in the root sequence. θ_{eq}: G+C equilibrium frequency of the simulation model.

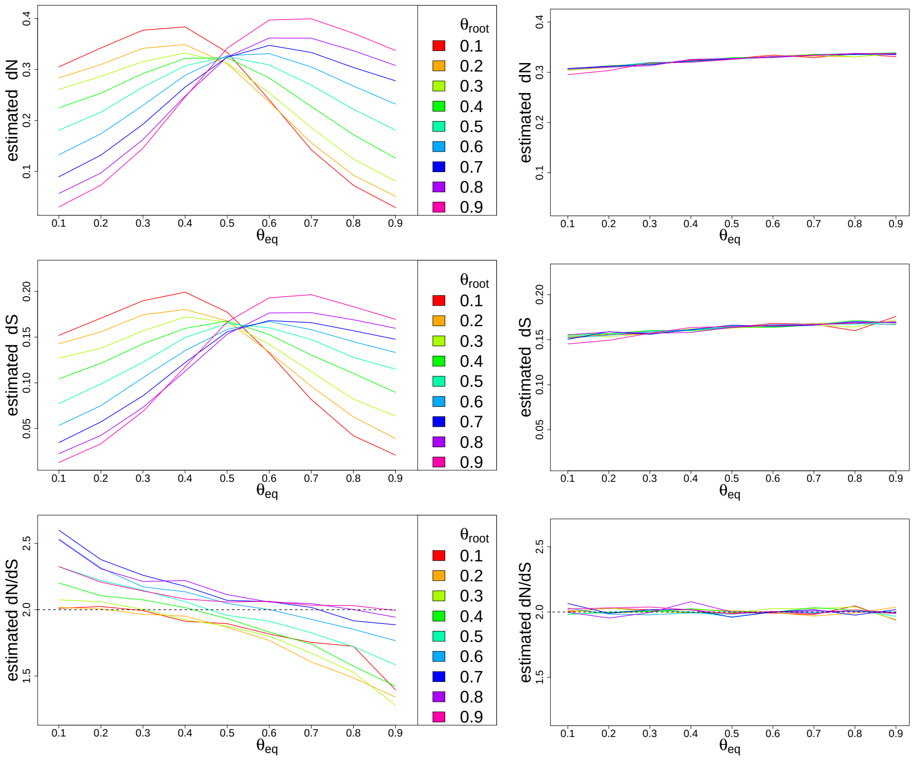

Figure S5: **Estimates of dN, dS and $\frac{dN}{dS}$** with a stationary model (left) and non-stationary model (right), on simulated data with changing G+C content and $\omega = 2$. θ_{root}: G+C frequency in the root sequence. θ_{eq}: G+C equilibrium frequency of the simulation model.

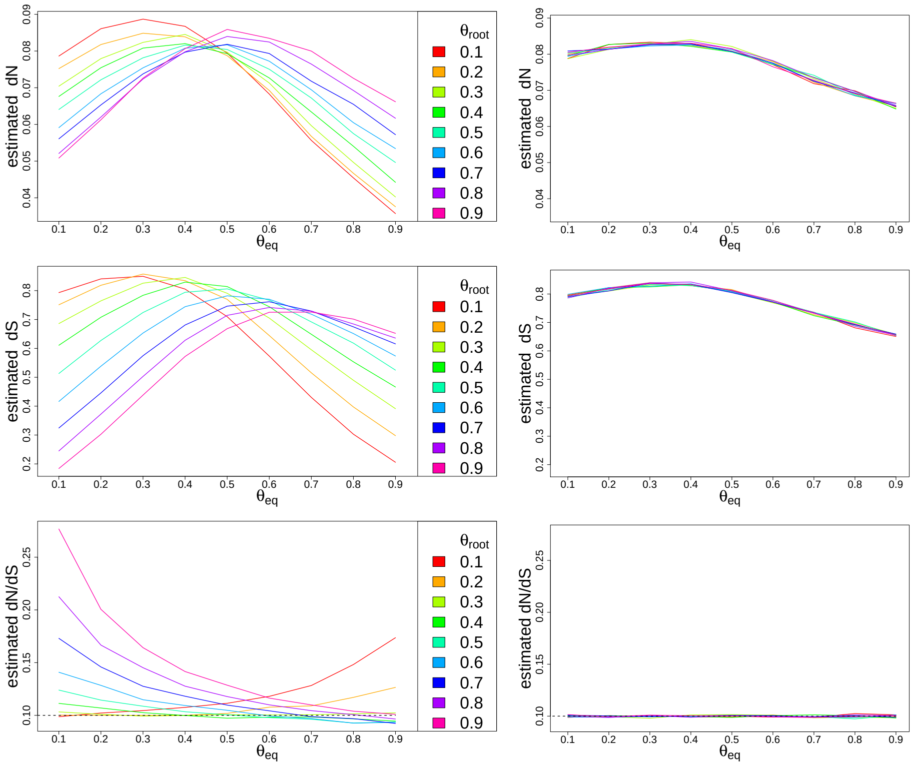

Figure S6: **Estimates of dN, dS and $\frac{dN}{dS}$** with a stationary model (left) and non-stationary model (right), on simulated data with changing G+C content in codon position 1, and $\omega = 0.1$. θ_{root}: G+C frequency in codon position 1 of the root sequence. θ_{eq}: G+C equilibrium frequency in codon position 1 of the simulation model.

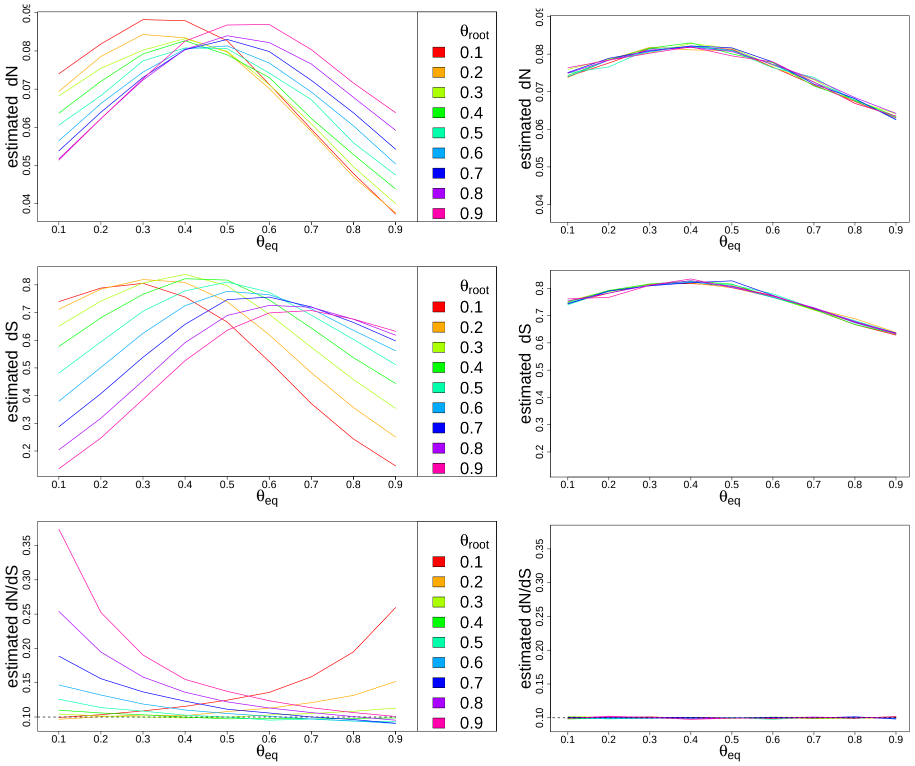

Figure S7: **Estimates of dN, dS and $\frac{dN}{dS}$** with a stationary model (left) and non-stationary model (right), on simulated data with changing G+C content in codon position 2, and $\omega = 0.1$. θ_{root}: G+C frequency in codon position 2 of the root sequence. θ_{eq}: G+C equilibrium frequency in codon position 2 of the simulation model.

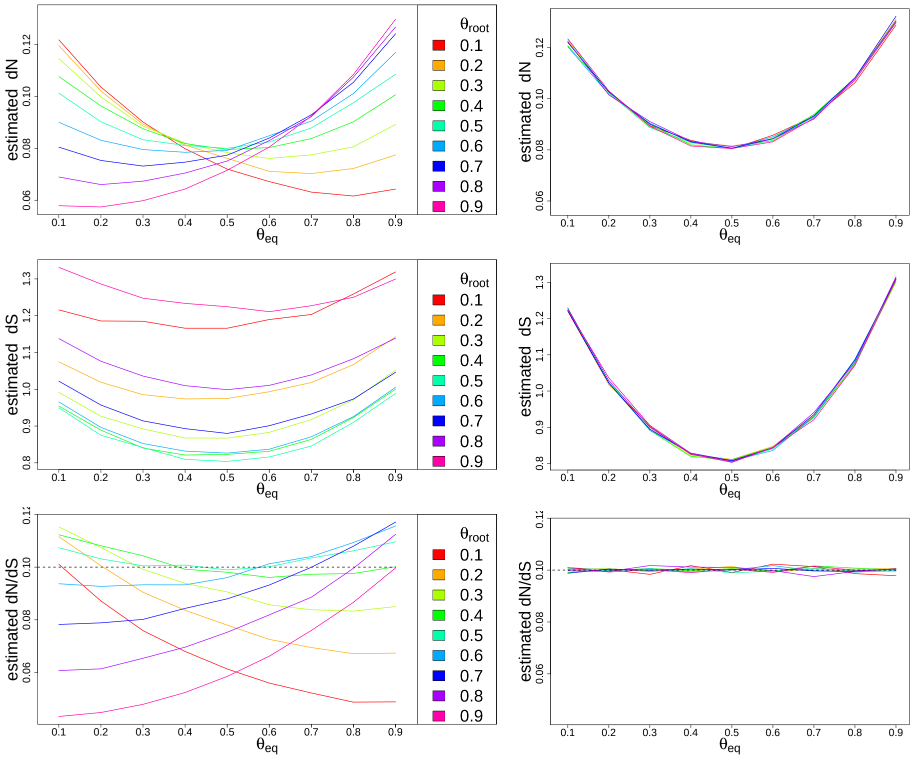

Figure S8: **Estimates of dN, dS and $\frac{dN}{dS}$** with a stationary model (left) and non-stationary model (right), on simulated data with changing G+C content in codon position 3, and $\omega = 0.1$. θ_{root}: G+C frequency in codon position 3 of the root sequence. θ_{eq}: G+C equilibrium frequency in codon position 3 of the simulation model.

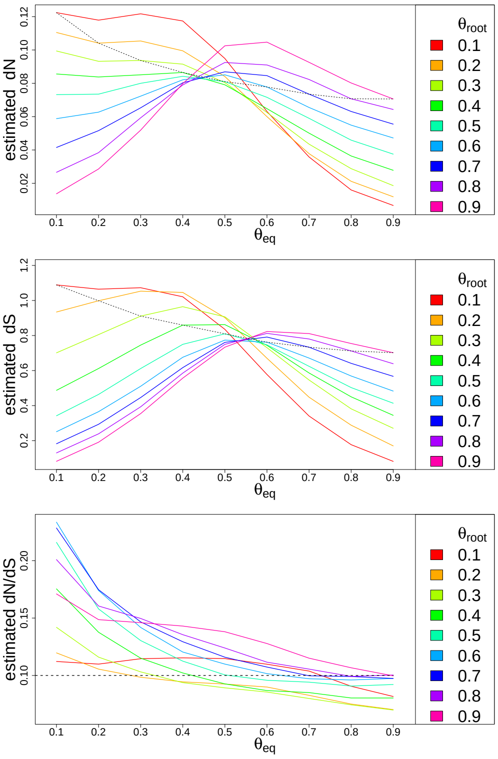

Figure S9: **Substitution rates estimated with codeml**. Sequences were simulated with changing G+C content and $\omega = 0.1$. From top to bottom: dN, dS and $\frac{dN}{dS}$. θ_{root}: G+C frequency in the root sequence. Dashed curve : estimates on sequences at equilibrium. θ_{eq}: G+C equilibrium frequency of the simulation model.

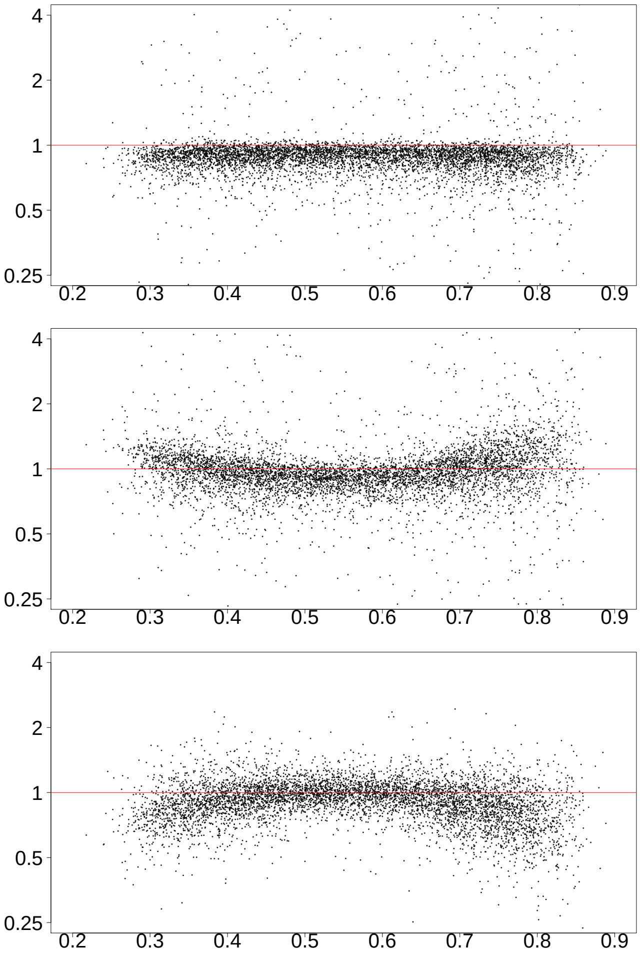

Figure S10: **log2 of the ratios of estimates of dN, dS and dN/dS** with a stationary model over the estimates with a non-stationary model, in function of human $GC3$ content.

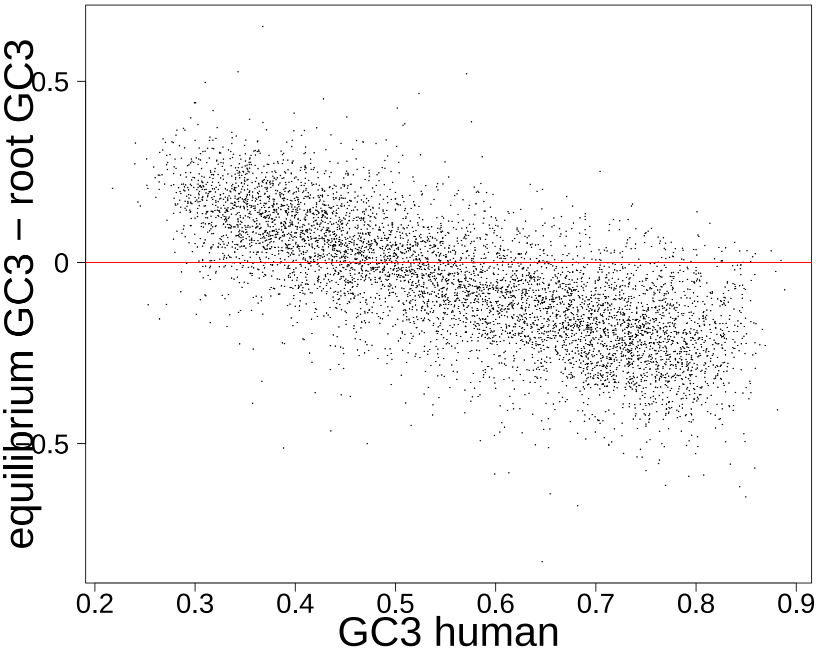

Figure S11: **Estimated difference between equilibrium GC3 in primate clade and root GC3** compared to observed human GC3.
